# Commentary: The Alleged Coupling-Constitution Fallacy and the Mature Sciences

**DOI:** 10.3389/fpsyg.2016.02033

**Published:** 2016-12-27

**Authors:** Luke Kersten

**Affiliations:** Department of Philosophy, University of EdinburghEdinburgh, UK

**Keywords:** terminological issues, cognitive systems, problem solving, the extended mind, underdetermination

A commentary on **The Alleged Coupling-Constitution Fallacy and the Mature Sciences** *by Ross, D., and Ladyman, J. (2010). The Extended Mind, ed R. Menary (Cambridge, MA: MIT Press), 155–166. ISBN: 9780262014038.*

The extended mind thesis holds that the mind can leak out into the world (Clark and Chalmers, 1998; Wilson, 2004; Clark, 2008). Ross and Ladyman (2010) have offered a novel critique of this view by arguing that the question of cognitive extension is largely terminological or semantic, that it is a product of an immature science employing unhelpful metaphors about containment. They write, for instance: “*We do not believe there is any basis for a general fact of the mater about what is and what isn't a cognitive system. Modelers will and should draw system boundaries in whichever ways maximize efficient capture of local phenomena*” (Ross and Ladyman, 2010, p. 156). There is no matter of fact as to whether cognitive systems are extended or not, as such descriptions are drawn from too early a stage of scientific investigation. In this commentary, I offer one response to Ross and Laydman's terminological or semantic objection to the extended mind.

Ross and Laydman's challenge can be framed as follows. If it turns out that calling cognitive systems extended or not is a matter of choice, then cognitive science can continue business-as-usual. However, if the extended mind thesis turns out to offer principled grounds for talking about cognitive systems, then some of the most basic assumptions of cognitive science might require revision—for example, that the brain is the seat of cognition (Wilson and Clark, 2009). Thus, in order to evaluate the merits of the extended mind thesis, it first needs to be shown that there is a substantive issue at stake.

To gain traction on the challenge, consider how an issue is normally identified as semantic rather than substantive. Consider, for example, the simple case of how to pronounce the word for the red fruit-berry of the nightshade plant *Solanum lycopersicum*, commonly referred to as a tomato. Those in North America might say “toma/to” [təˈmeɪˌto℧], while those in the United Kingdom might say “to/ma/to” [təˈmα:tə℧]. Under normal circumstances, it seems wrongheaded to ask whether one or the other pronunciation is correct, even though the intension and extension are the same. Contrast this with whether the basic subatomic elements in physics are particles or waves. Here, the choice of terms seems more principled. There seems to be something about the world that determines which term is more suitable, even though the current state of the science is undecided. What accounts for the difference arguably is that in the latter's case the choice among terms has something to do with how the terms track matters of fact.

One philosophical manifestation of this idea is “underdetermination,” the idea that there might be two or more equally good theories or explanations for some phenomena (Laudan, 1990; Laudan and Leplin, 1991). The classic example is that a high correlation of cartoon viewing and violent playground behavior equally supports the explanations that watching cartoons causes violent children and that children prone to violence tend to enjoy watching cartoons. Because there are multiply sufficient methods of description, the evidence alone fails to determine the correct view. The choice among explanations is underdetermined. The question is whether such a state of affairs holds in the case of cognition. Are cognitive systems underdetermined such that the choice among labels is relative? Or, is there is a matter of fact determining whether the extended or internalist description is more appropriate?

Consider a particular cognitive task: problem solving—to be more specific, consider how cognizers might solve a particular board configuration of the board game Rush Hour (the purpose of which is to move small toy vehicles across a board). One method is that agents might form internal representations and then manipulate those representations to solve the problem. Another is that agents might repeatedly use visual inspection, neural processing and actual movements of cars on the board to solve the problem. The first of these methods corresponds to the traditional explanation offered in cognitive science, where vision provides the initial input to an internal cognitive system which then executes bodily actions in service of solving some particular task (Newell et al., 1960; Newell and Simon, 1972).

Emerging research, however, suggests that people often solve problems more in line with the second method. Cognizers often rely on repeated action-perception cycles during problem solving (Kirsch, 2009). Repeated interactions and transformations of the physical environment—for example, configurations of the board—are used to literally see different pathways through the problem space. Notice the difference in the underlying cognitive systems. In the first method the cognitive system is internally bounded and trades in internal representations, while in the second method the cognitive system is, in part, constituted by the perceptual and motor interactions integratively coupled to the internal processes. The second method employs a cognitive system that spreads out across brain, body, and world.

One might respond by arguing that something has been smuggled into the foregoing analysis to bias an extended conclusion. Notice, however, that the method for identifying the cognitive system was task neutral. First, the function of interest was specified. Then, the supporting system was individuated, which revealed causally integrated units spread across the brain, body, and world. The relevant system turned out to be extended only after the cognitive task and function were specified, even though it could have turned out to be entirely internal.

Return, then, to the initial worry about terminological relativeness from Ross and Ladyman (2010). It was said that an issue was semantic when it could not be shown that there was a matter of fact for deciding between competing accounts. What has been shown is that, in some cases, such a requirement fails to obtain. There is a fact of the matter as to whether cognitive systems are extended in at least some cases. The extended mind debate, then, is more than a mere terminological or semantic issue. This is not to say that the extended mind thesis is true, but it is to say that contrary to Ross and Ladyman (2010) the question of the extended mind is a substantive one.

## Author contributions

The author confirms being the sole contributor of this work and approved it for publication.

### Conflict of interest statement

The author declares that the research was conducted in the absence of any commercial or financial relationships that could be construed as a potential conflict of interest.
